# Synthesis of New Visnagen and Khellin Furochromone Pyrimidine Derivatives and Their Anti-Inflammatory and Analgesic Activity

**DOI:** 10.3390/molecules16031956

**Published:** 2011-02-25

**Authors:** Ameen Ali Abu-Hashem, Mohamed M. Youssef

**Affiliations:** 1 Departments of Photochemistry, National Research Center, 12622 Dokki, Giza, Egypt; 2 Department of Chemistry, Faculty of Science, Cairo University, Giza, Egypt

**Keywords:** khellin, visnagen, furochromones, anti-inflammatory and analgesic activity

## Abstract

6-[(4-Methoxy/4,9-dimethoxy)-7-methylfurochromen-5-ylideneamino]-2-thioxo-2,3-dihydropyrimidin-4-ones **1a,b** were prepared by reaction of 6-amino-2-thiouracil with visnagen or khellin, respectively. Reaction of **1a,b** with methyl iodide afforded furochromenylideneaminomethylsulfanylpyrimidin-4-ones **2a,b**. Compounds **2a,b** were reacted with secondary aliphatic amines to give the corresponding furochromen-ylideneamino-2-substituted pyrimidin-4-ones **3a-d**. Reaction of **3a-d** with phosphorus oxychloride yielded 6-chlorofurochromenylidenepyrimidinamines **4a-d**, which were reacted with secondary amines to afford furochromenylideneamino-2,6-disubstituted pyrimidin-4-ones **5a-d**. In addition, reaction of **5a-d** with 3-chloropentane-2,4-dione gave 3-chloro-furochromenylpyrimidopyrimidines **6a-d**. The latter were reacted with piperazine and morpholine to give 1-(furochromenyl)-pyrimidopyrimidine-3,6,8-triylpiperazines or-3,6,8-triylmorpholines **7a-d**. The chemical structures of the newly synthesized compound ware characterized by IR, ^1^H-NMR, ^13^C-NMR and mass spectral analysis. These compounds were also screened for their analgesic and anti-inflammatory activities. Some of them, particularly **3-7**, exhibited promising activities.

## 1. Introduction

**Figure 1:**
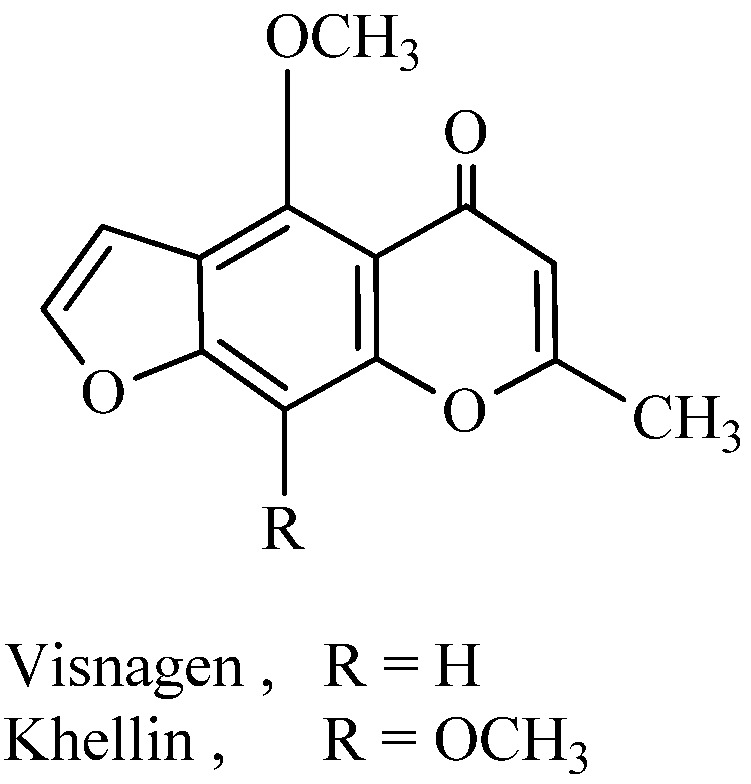
Chemical structure of khellin and visnagen.

Khellin and visnagen, 4,9-dimethoxy- or 4-methoxy-7-methyl-furo[3,2-*g*]chromen-5-one, respectively (Figure 1) are obtained from fruits and seeds of the plant *Ammi visnaga*. The khellin and visnagen extract has been widely employed as a herbal medicine in the treatment of angina [1]. Khellin is used as a spasmolytic agent and for kidney stone treatment [2]. Khellin and visnagen extract significantly prolongs the induction time of nucleation of calcium oxalate [3]. Khellin has been used for photochemotherapeutic treatment of vitiligo and psoriasis [4] and the photodynamic properties of khellin and visnagen in their photoreaction with DNA have been studied [5]. Recently, khellin was proved to have phototherapeutic properties that are similar to those of the psoralens, but with substantially lower phototoxic effects and DNA mutation effects. Its penetration into the hair follicles is enhanced by encapsulating it into liposomes. Subsequent activation of khellin with UV light stimulates the melanocytes in the hair follicles [6]. The fact that furochromone derivatives are known to have anti-inflammatory properties [7] and analgesic properties [8], prompted us to synthesize and investigate such properties in khellin and visnagen derivatives as typical furochromones.

From a photobiological point of view, furochromones show valuable phototoxicity toward various kinds of microorganisms and also valuable genotoxic activity on various biological substrates [9,10]. In the present work, we planned the synthesis of various furochromenylidenylpyrimidine derivatives representing new heterocyclic compounds. These compounds were also screened for theiranti-inflammatory and analgesic activities.

## 2. Results and Discussion

**Scheme 1:**
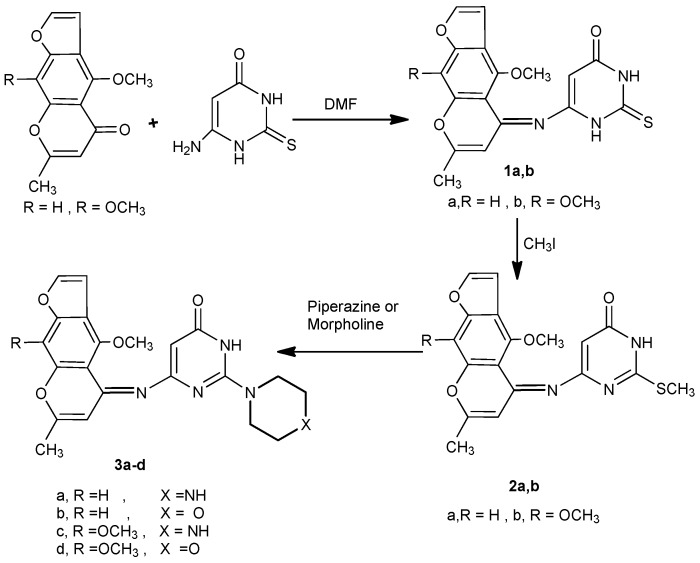
Condensation of khellin and visnagen with aminothiouracil, methylation and reaction with secondary amines.

Condensation of visnagen and khellin with 6-amino-2-thiouracil in dimethylformamide [11,12] yielded the 6-(4-methoxy/4,9-dimethoxy)-7-methylfuro[3,2-*g*]chromen-5-ylideneamino)-2-thioxo-2,3-dihydro-*1H*-pyrimidin-4-ones **1a,b**. The IR spectra of compounds **1a,b** revealed the presence of only one carbonyl band, in addition to the absence of the amino group band. The mass spectra of **1a** and **1b** showed molecular ion peaks at *m/z* 355 (96%) and 385 (90%), respectively. Compounds **1a,b** underwent alkylation at the sulphur atom upon treatment with methyl iodide in aqueous ethanolic KOH solution, to afford 6-((4-methoxy/4,9-dimethoxy)-7-methylfuro[3,2-g]chromen-5-ylidene-amino)-2-methylsulfanyl-3*H*-pyrimidin-4-ones **2a,b**. The ^1^H-NMR spectra of **2a** and **2b** showed singlets at δ 2.68 and 2.65 ppm, respectively, corresponding to a SCH_3_ group. Reaction of the2-methylsulfanyl derivatives **2a,b** with secondary aliphatic amines, namely piperazine and morpholine, in methanol [13] produced the 6-(4-methoxy/4,9-dimethoxy)-7-methylfuro[3,2-g]chromen-5-ylideneamino)-2-(piperazin/morpholin)-1-yl-3*H*-pyrimidin-4-ones **3a-d** (Scheme 1).

Moreover, the reaction of **3a-d** with phosphorus oxychloride in dry dioxane [14] afforded the (6-chloro-2-(piperazin/morpholin)-1-yl-pyrimidin-4-yl)-(4-methoxy/4,9-dimethoxy)-7-methylfuro[3,2-g]-chromen-5-ylidene)amines **4a-d**. The IR spectra of **4a-d** revealed the absence of any absorption bands in the NH and carbonyl regions. Compounds **4a-d**, having an active chlorine substituent, reacted with either piperazine or morpholine in boiling methanol to produce 2,6-(dipiperazin/dimorpholin)-1-yl-pyrimidin-4-yl)-(4-methoxy/4,9-dimethoxy)-7-methylfuro[3,2-g]-chromen-5-ylidene)amines **5a-d**. The structures of compounds **5a-d** were confirmed by their correct elemental analyses values, as well as compatible spectral data (see Experimental). Compounds **5a-d** reacted with 3-chloropentane-2,4-dione (a typical β-diketone) in acetic acid, in the presence of zinc dust, to afford the corresponding 3-chloro-1-(4-methoxy/4,9-dimethoxy)-7-methyl-5*H*-furo[3,2-g]chromen-5-yl)-2,4-dimethyl-6,8-dipiperazin/di-morpholin)-1-yl-1,3,4,6-tetrahydro-2*H*-pyrimido[1,6-a]pyrimidines **6a-d** in good yield. Formation of **6a-d** from the corresponding **5a-d** may proceed by initial reduction of compounds **5a-d**, followed by cyclocondensation of the intermediates produced with the diketone followed by a necessary final reduction step to produce **6a-d**.

**Scheme 2:**
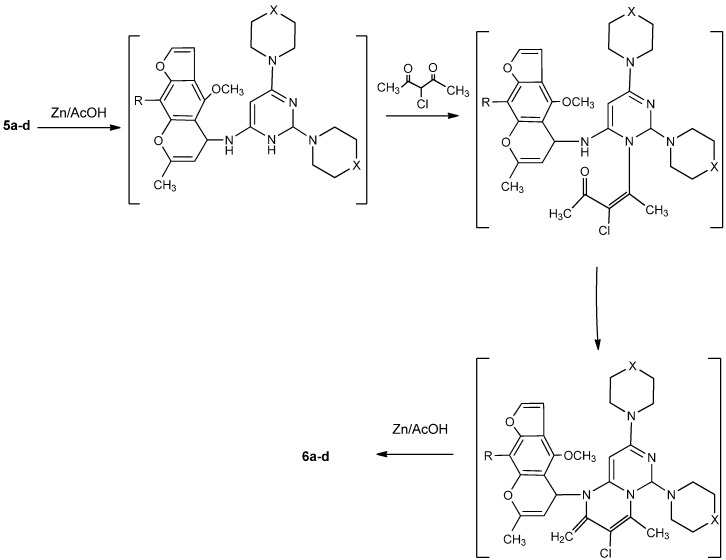
Suggested mechanism for the formation of **6a-d** from **5a-d**.

**Scheme 3:**
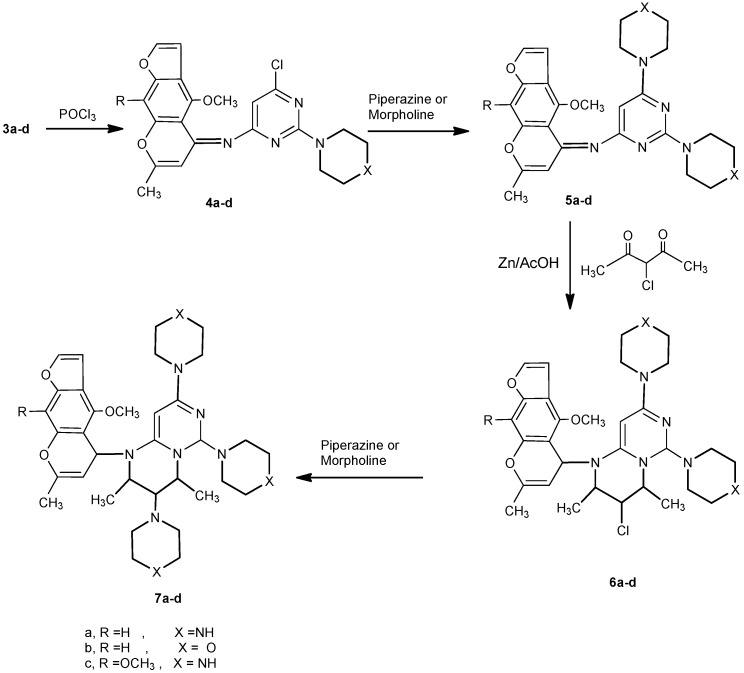
Chlorination of **3a-d**, condensation with amines and cyclocondensation with β-diketone.

The steps of the suggested mechanism are shown in Scheme 2. The ^1^H-NMR spectrum of **6a**, for example, showed a doublet at 1.15 ppm, a multiplet at 3.14 and a triplet at 3.92, which support the proposed reduced structure **6**. Finally, the 3-chlorofurochromenepyrimido/pyrimidines reacted with either piperazine or morpholine in boiling methanol to give the corresponding 3,6,8-tripiperazin-1-yl or 3,6,8-trimorpholin-1-yl derivatives **7a-d** (Scheme 3). Compatible analytical and spectral data were obtained for compounds **7a-d** (see Experimental).

### 2.1. Anti-Inflammatory Activity

The anti-inflammatory activity was evaluated in rats by the carrageenan-induced paw edema test. The data (Table 1) indicated that all the tested compounds protected rats from carrageenan-induced inflammation, and that some of the tested compounds (**6a-d** and **7a-d**) are more potent than our previously reported ones [15,16]. Compounds **2-5** showed similar and higher anti-inflammatory activity than diclofenac sodium.

**Table 1:** Percent anti-inflammatory activity of the tested compounds (carrageenan-induced paw edema test in rats).

Compd. No.	Percent protection		
	1 hour	2 hours	3 hours
Visnagen	38.6 ± 1.38 *	40.2 ± 1.39 *	26.2 ± 1.29 *
Khellin	40.3 ± 1.40 *	41.5 ± 1.42 *	30.4 ± 1.32 *
**1a**	41.5 ± 1.35 *	40.4 ±1.36 *	28.2 ± 1.28 *
**1b**	42.6 ± 1.45 *	41.5 ±1.28 *	30.1 ± 1.30 *
**2a**	44.5 ± 1.28 *	43.6 ±1.24 *	33.1 ± 1.01 *
**2b**	45.7 ± 1.30 *	44.8 ±1.28 *	34.2 ± 1.02 *
**3a**	52.6± 1.10 **	50.8 ± 1.30 *	39.4± 1.25 *
**3b**	48.6 ± 1.40 *	45.6 ±1.40 *	35.1 ± 1.04 *
**3c**	52.8± 1.12 **	52.6 ± 1.10 *	39.6± 1.23 *
**3d**	49.9 ± 1.50 **	48.8 ±1.42 *	36.5 ± 1.20 *
**4a**	54.4± 1.23 **	53.6 ± 1.22 *	40.4± 1.04 *
**4b**	53.8± 1.20 **	52.6 ± 1.10 *	40.1± 1.02 *
**4c**	55.3 ± 1.25 **	54.8 ± 1.24 *	40.6± 1.05 *
**4d**	54.1± 1.22 **	53.2 ± 1.20 *	40.2± 1.03 *
**5a**	57.9 ± 1.39 **	58.9 ± 1.30 *	41.5 ± 1.08 *
**5b**	57.3 ± 1.37 **	58.8 ± 1.29 *	41.3± 1.06 *
**5c**	58.4 ± 1.40 **	59.1 ± 1.31 **	42.1 ± 1.23 *
**5d**	57.6 ± 1.38 **	60.0 ± 1.68 **	41.4 ± 1.07 *
**6a**	59.7 ± 1.45 **	59.5 ± 1.35 **	42.8 ± 1.26 *
**6b**	59.4 ± 1.05 **	59.1 ± 1.31 **	42.2 ± 1.24 *
**6c**	59.8 ± 1.52 **	59.7 ± 1.45 **	45.7 ± 1.52 *
**6d**	59.6 ± 1.41 **	59.2 ± 1.32 **	42.4 ± 1.25 *
**7a**	61.8± 1.88 **	61.4 ± 1.74 **	48.2 ± 1.65 *
**7b**	60.5± 1.80 **	60.2 ± 1.62 **	46.6 ± 1.60 *
**7c**	62.3± 1.92 **	61.6 ± 1.78 **	48.5 ± 1.68 *
**7d**	60.9± 1.85 **	60.4 ± 1.65 **	47.1 ± 1.63 *
Control	6.3 ± 0.26	5.6 ± 0.40	3.4 ± 0.96
Diclofenac Sodium	52.6 ± 0.96 *	60.5± 1.55 **	42.2 ± 1.39 *

Each value represents the mean ± S.E (n = 6).Significance levels * p < 0.5, ** p < 0.001 as compared with respective control. Dose (20 mg/kg). For the selected tested compound.

### 2.2. Analgesic Activity

The analgesic activity was determined by the hot-plate test (central analgesic activity) and acetic acid induced writhing assay. The results (Table 2 and Table 3) revealed that all tested compounds exhibited significant activity. Most of the tested compounds have nearly the same activity as the reference drug and the remaining tested compound have good activities in central analgesic activity. Also compound **7c** exhibited activities higher than the reference.

**Table 2:** Central analgesic activity (Hot plate test).

Group	Reaction time (min)			
	0 min	30 min	60 min	90 min
Control	8.25 ± 0.35	8.20 ± 0.36 b	8.75 ± 0.55 b	9.80 ± 0.48 b
Visnagen	5.40 ± 0.56	6.30 ± 0.50	8.90 ± 0.60	9.80 ± 0.25 a
Khellin	5.50 ± 0.58	6.50 ± 0.40	9.01 ± 0.70	10.01 ± 0.22 a
**1a**	5.60 ± 0.30	6.70± 0.43	9.25 ± 0.55	10.10 ± 0.20a
**1b**	6.10 ± 0.20	7.30 ± 0.10 b	7.40 ± 0.10 a	10.20 ± 0.25 a
**2a**	6.20 ± 0.10	7.45 ± 0.20 b	8.10± 0.20 a	10.50 ± 0.30 a
**2b**	6.40 ± 0.20	7.55 ± 0.25 b	8.25± 0.30 a	10.80 ± 0.35 a
**3a**	7.01 ± 0.30	8.10 ± 0.40	10.55 ± 0.50 a	10.90 ± 0.60 a b
**3b**	6.65 ± 0.10	7.90 ± 0.20 b	8.45 ± 0.50 a	11.20 ± 0.30 a
**3c**	7.10 ± 0.10	8.20 ± 0.30 a	9.50 ± 0.25 a	10.55 ± 0.10 a b
**3d**	6.65 ± 0.10	7.90 ± 0.20 b	8.45 ± 0.50 a	11.20 ± 0.30 a
**4a**	7.85 ± 0.35	8.60 ± 0.40 a	10.01 ± 0.20a	11.05± 0.40 a b
**4b**	7.30 ± 0.20	8.30 ± 0.40 a	9.80 ± 0.30 a	10.80 ± 0.20 a b
**4c**	8.10 ± 0.20	8.80 ± 0.25 a	10.10 ± 0.25 a	11.10 ± 0.30 b
**4d**	7.40 ± 0.30	8.50 ± 0.30 a	9.90 ± 0.35 a	10.90 ± 0.30 a b
**5a**	8.75 ± 0.60	9.10 ± 0.50 a	10.70 ± 0.40 a	11.50 ± 0.40 b
**5b**	8.30 ± 0.30	8.90 ± 0.30 a	10.30 ± 0.20 a	11.20 ± 0.25 b
**5c**	8.95 ± 0.68	9.20 ± 0.60 a	10.90 ± 0.50 a	11.60 ± 0.30 b
**5d**	8.55 ± 0.50	9.01 ± 0.40 a	10.50 ± 0.30 a	11.40 ± 0.30 b
**6a**	9.30 ± 0.30	9.40 ± 0.30 a	10.40 ± 0.25 a	11.10± 0.30 a b
**6b**	9.10 ± 0.30	9.20 ± 0.40 a	10.20 ± 0.20 a	11.10 ± 0.40a b
**6c**	9.40 ± 0.40	9.50 ± 0.30 a	10.50 ± 0.20 a	11.20± 0.35 a b
**6d**	9.20 ± 0.20	9.30 ± 0.10 a	10.30 ± 0.30 a	11.01± 0.20 a b
**7a**	9.70 ± 0.40	9.80 ± 0.55 a	10.75 ± 0.35 a	11.44± 0.44 a b
**7b**	9.50 ± 0.20	9.60 ± 0.40 a	10.60 ± 0.25 a	11.30± 0.30 a b
**7c**	9.80 ± 0.50	9.95 ± 0.65 a	10.85 ± 0.45 a	11.98 ± 0.88 a b
**7d**	9.60 ± 0.30	9.70 ± 0.45 a	10.65 ± 0.30 a	11.40± 0.40 a b
Diclofanc sodium	6.50 ± 0.45	10.05 ± 0.15 a	11.40 ± 0.55 a	13.18 ± 0.40 a

Values represent the mean ± S.E. of six animals for each groups. a. P < 0.05: Statistically significant from Control. (Dunnett’s test). b. P < 0.05: Statistically significant from ASA. (Dunnett’s test).* Significant at P < 0.0.

**Table 3:** Percent analgesic activity (peripheral, writhing test).

Compd. No.	Percent protection			
	30 min	1 hours	2 hour	3 hours
Visnagen	39.20 ± 1.20 *	44 ± 1.10 *	48.1 ± 1.70	32.20 ± 1.25 *
Khellin	40.7 ± 1.65 *	46 ± 1.35 *	49.4 ± 1.70	33.9 ± 1.10 *
**1a**	42.6 ± 1.40 *	52 ± 1.25 *	50.4 ± 1.60	35.8 ± 1.39 *
**1b**	45.0 ± 1.90 *	53 ± 1.40 *	55.6 ± 1.38	36.3 ± 1.20 *
**2a**	46.5 ± 1.50 *	50 ± 1.10 **	52.2 ± 1.30	37.5 ± 1.30 *
**2b**	48.2 ± 1.55 *	52 ± 1.20 *	54.3 ± 1.25	38.7 ± 1.40 *
**3a**	54.6 ± 1.40 *	58 ± 1.25 *	62.6 ± 1.20 *	46.4 ± 1.10 *
**3b**	50.3 ± 1.35 *	54 ± 1.10 *	56.4 ± 1.35	40.5 ± 1.35 *
**3c**	55.8 ± 1.50 *	59 ± 1.20 *	63.6 ± 1.30 *	47.3 ± 1.30 *
**3d**	52.6 ± 1.35 *	56 ± 1.05 *	58.6 ± 1.35	42.1 ± 1.40 *
**4a**	60.4 ± 1.40 **	63 ± 1.50 **	67.7± 1.70 **	48.4 ± 1.55 *
**4b**	58.5 ± 1.20 *	60 ± 1.30 *	65.4 ± 1.55*	46.5 ± 1.35 *
**4c**	61.5 ± 1.50 **	64 ± 1.55 **	68.8 ± 1.80 **	49.5 ± 1.60 *
**4d**	59.6 ± 1.30 *	62 ± 1.40 *	66.1 ± 1.60 *	47.3 ± 1.50 *
**5a**	66.50 ± 1.20 **	68 ± 1.55 **	70.6 ± 1.30 **	54.5 ± 1.10 *
**5b**	62.50 ± 1.40 **	65 ± 1.35 **	69.10 ±1.25 **	52.1 ± 1.30 *
**5c**	68.10 ± 1.25 **	70 ± 1.85 **	71.8 ± 1.35 **	55.4 ± 1.05 *
**5d**	65.40 ± 1.10 **	67 ± 1.50 **	70.20 ±1.15 **	53.2 ± 1.20 *
**6a**	69.60 ± 1.55 **	72 ± 1.90 **	74.3 ± 1.50 **	58.8 ± 1.20 *
**6b**	69.1 ± 1.45 **	70 ± 1.80 **	72.6 ± 1.40 **	56.3 ± 1.10 *
**6c**	69.80 ± 1.60 **	73 ± 1.95 **	75.5 ± 1.55 **	59.8 ± 1.19 *
**6d**	69.4 ± 1.50 **	71 ± 1.85 **	73.5 ± 1.45 **	57.5 ± 1.15 *
**7a**	74.4 ± 1.10 **	77.5 ± 1.30 **	78.3 ± 1.20	64.4 ± 1.25 **
**7b**	70.1 ± 1.01 **	75.3 ± 1.50 **	76.5 ± 1.25	62.4 ± 1.20 **
**7c**	75.6 ± 1.20 **	78.4 ± 1.40 **	79.5 ± 1.35	65.6 ± 1.30 **
**7d**	72.2 ± 1.05 **	76.2 ± 1.45 **	77.6 ± 1.30	63.5 ± 1.35 **
Control	02.0 ± 0.36	05.0 ± 0.50	04.0 ± 0.58	04.0 ± 0.90
Diclfenac sodium	45.0 ± 0.96 *	54.3 ± 1.18 *	61 ± 1.50 *	38 ± 1.14 *

Each value represents the mean ± S.E (n = 6).Significance levels * p < 0.5, ** p < 0.001 as compared with respective control. Dose (20 mg/kg). For the selected tested compound .Drug in peripheral analgesic activity testing. The remaining compounds have the same activity in Peripheral analgesic activity testing.

## 3. Conclusions

The new ring systems prepared seem to be interesting for biological activity studies. Furthermore, the present investigation offers rapid and effective new procedures for the synthesis of the poly-condensed new heterocyclic ring systems. Compounds **6a-d** and **7a-d** exhibited potent anti-inflammatory and analgesic activities. It is worth mentioning that the incorporation of methoxy, dimethoxy, -furo[3,2-g]chromen, di- and tri-(piperazine or morpholine) and tetrahydropyrimido[1,6-a]pyrimidine moieties resulted in significant anti-inflammatory and analgesic activities. In conclusion, we report herein a simple and convenient route for the synthesis of some new heterocyclic compounds based on furochromene pyrimidine derivatives for anti-inflammatory and analgesic evaluation.

## 4. Experimental

### 4.1. General

All melting points were taken on an Electrothermal IA 9100 series digital melting point apparatus (Shimadzu, Japan). Elemental analyses were performed at Vario EL (Elementar, Germany). Microanalytical data were processed in the microanalytical center, Faculty of Science, Cairo University and National Research Center. The IR spectra (KBr disc) were recorded using a Perkin-Elmer 1650 spectrometer (USA).^1^H-NMR spectra were determined using Jeol 270 MHz and Jeol JMS-AX 500 MHz (Jeol, Japan) spectrometers with Me_4_Si as an internal standard. Mass spectra were recorded on an EIMs-QP 1000 EX instrument (Shimadzu) at 70 eV. Pharmacological evaluations were done by the Pharmacology unit, Department of Pharmacognosy, Faculty of Pharmacy, Mansoura University, Egypt.

### 4.2. General Procedure for the Synthesis of 6-((4-Methoxy/4,9-dimethoxy)7-methylfuro[3,2-g]chromen-5-ylideneamino)-2-thioxo-2,3-dihydro-1H-pyrimidin-4-ones **1a,b**

A mixture of visnagen (2.3 g, 10 mmol) or khellin (2.6 g, 10 mmol) and 6-amino-2-thiouracil (1.43 g, 10 mmol), was refluxed in dimethylformamide (50 mL) for 6-8 h. The reaction mixture was cooled; the deposited precipitate was filtered off, washed with ethanol, dried, and recrystallized to obtain **1a,b** as crystalline products.

*6-(4-Methoxy-7-methylfuro[3,2-g]chromen-5-ylideneamino)-2-thioxo-2,3-dihydro-1H-pyrimidin-4-one* (**1a**). Obtained from visnagen as yellow crystals, m.p. 228-230 ºC, crystallized from methanol(80% yield); IR (KBr, cm^−1^): 3,390 (br, NHs), 3,030 (CH, aryl), 2,915 (CH, alkyl), 1,690 (CO), 1,630 (C=N); ^1^H-NMR: 1.75 (s, 3H, CH_3_), 3.75 (s, 3H, OCH_3_), 5.45 (s, 1H, H_pyran_), 6.69 (s, 1H, H_furan_), 6.74 (s, 1H, H_benzene_), 6.90 (s, 1H, H_pyrimidine_), 7.55 (s, 1H, H_furan_), 10.40, 11.60 (2 br s, 2NH, D_2_O exchangeable). ^13^C-NMR: 23.3, 56.4 (2C, CH_3_, OCH_3_), 93.5, 95.4, 99.7, 102.5, 108.4, 110.30, 147.1, 155.5, 157.4, 160.5, 162.3, 165.5, 166.3 (Ar-C), 169.2 (CO), 178.4 (CS). MS (70 eV, %) *m/z*, 355 (M^+^, 96%). Anal. Calc. for C_17_H_13_N_3_O_4_S (355.37); Requires (Found): C, 57.46 (57.52); H, 3.69 (3.62); N, 11.82 (11.88); S, 9.02 (9.15).

*6-(4,9-Dimethoxy-7-methylfuro[3,2-g]chromen-5-ylideneamino)-2-thioxo-2,3-dihydro-1H-pyrimidin-4-one* (**1b**). Obtained from khellin as yellowish crystals, m.p. 240-242 ºC, crystallized from dimethylformamide (82% yield); IR (KBr, cm^−1^): 3,391 (br, NHs), 3,031 (CH, aryl), 2,914 (CH, alkyl), 1,688 (CO), 1,631 (C=N). ^1^H-NMR: 1.74 (s, 3H, CH_3_), 3.76 (s, 6H, 2 OCH_3_), 5.46 (s, 1H, H_pyran_), 6.68 (s, 1H, H_furan_), 6.91 (s, 1H, H_pyrimidine_), 7.54 (s, 1H, H_furan_), 10.42, 11.62 (2 br s, 2NH, D_2_O exchangeable). MS (70 eV, %) *m/z* 385 (M^+^, 90%). Anal. Calc. (Found) for C_18_H_15_N_3_O_5_S (385.39): C, 56.10 (56.20); H, 3.92 (3.98); N, 10.90 (10.85); S, 8.32 (8.37).

### 4.3. General Procedure for the Synthesis of 6-((4-Methoxy/4,9-dimethoxy)-7-methylfuro[3,2-g]chromen-5-ylideneamino)-2-methylsulfanyl-3H-pyrimidin-4-ones **2a,b**

To a warmed ethanolic KOH solution (prepared by dissolving 0.01 mol of KOH in 50 mL ethanol) was added each of **1a** (3.55 g, 10 mmol), or **1b** (3.85 g, 10 mmol), heating was continued for 30 min and the mixture was allowed to cool to room temperature, and methyl iodide (12 mmol) was added. The mixture was stirred under reflux for 6 hours, then cooled to room temperature and poured into cold water (100 mL). The solid product that precipitated was filtered off, washed with 100 mL water; the product was dried and crystallized to produce **2a,b**.

*6-(4-Methoxy-7-methylfuro[3,2-g]chromen-5-ylideneamino)-2-methylsulfanyl-3H-pyrimidin-4-one* (**2a**). Obtained from **1a** as white crystals, m.p. 275-277 ºC, crystallized from dioxane (75% yield); IR (KBr, cm^−1^): 3,385 (br, NH), 3,032 (CH, aryl), 2,918 (CH, alkyl), 1,686 (CO), 1,629 (C=N); ^1^H-NMR: 1.73 (s, 3H, CH_3_), 2.68 (s, 3H, SCH_3_), 3.75 (s, 3H, OCH_3_), 5.50 (s, 1H, H_pyran_), 6.67 (s, 1H, H_furan_), 6.72 (s, 1H, H_benzene_), 6.93 (s, 1H, H_pyrimidine_), 7.53 (s, 1H, H_furan_), 10.20 (brs, NH, D_2_O exchangeable).MS (70 eV, %) *m/z* 369 (M^+^, 92%). Anal. Calc. (Found) for C_18_H_15_N_3_O_4_S (369.39): C, 58.53 (58.50); H, 4.09 (4.14); N, 11.38 (11.45); S, 8.68 (8.73). 

*6-(4,9-Dimethoxy-7-methylfuro[3,2-g]chromen-5-ylideneamino)-2-methylsulfanyl-3H-pyrimidin-4-one* (**2b**). Obtained from **1b **as a white powder, m.p. 293-295 ºC, crystallized from methanol (70% yield); IR (KBr, cm^−1^): 3,384 (br, NH), 3,030 (CH, aryl), 2,919 (CH, alkyl), 1,689 (CO), 1,630 (C=N); ^1^H-NMR: 1.75 (s, 3H, CH_3_), 2.65 (s, 3H, SCH_3_), 3.77 (s, 6H, 2 OCH_3_), 5.49 (s, 1H, H_pyran_), 6.68(s, 1H, H_furan_), 6.89 (s, 1H, H_pyrimidine_), 7.52 (s, 1H, H_furan_), 10.15 (br s, NH, D_2_O exchangeable).^13^C-NMR: 19.5, 23.2, 56.6, (4C, CH_3_, S-CH_3_, 2OCH_3_), 99.7, 101.5, 107.4, 109.2, 112.50, 127.1, 140.3, 145.1, 146.1, 149.2, 156.5, 157.5, 161.5, 165.3 (Ar-C), 169.4 (CO). MS (70 eV, %) *m/z* 399(M^+^, 91%). Anal. Calc. (Found) for C_19_H_17_N_3_O_5_S (399.42): C, 57.13 (57.20); H, 4.29 (4.25); N, 10.52 (10.57); S, 8.03 (8.18).

### 4.4. General Procedure for the Synthesis of 6-((4-Methoxy/4,9-dimethoxy)-7-methylfuro[3,2-g]chromen-5-ylideneamino)-2-(piperazin/morpholin)-1-yl-3H-pyrimidin-4-ones **3a-d**

To a warm solution of **2a** (3.69 g, 10 mmol) or **2b** (3.99 g, 10 mmol) in methanol (100 mL) was added the freshly distilled secondary aliphatic amines (piperazine and morpholine, 10 mmol). The reaction mixture was stirred under reflux for 8 h, and then allowed to cool to 0 ºC for 12 h. The solid obtained was filtered, washed with water (100 mL), dried, and crystallized from the appropriate solvent to produce **3a-d**.

*6-(4-Methoxy-7-methylfuro[3,2-g]chromen-5-ylideneamino)-2-piperazin-1-yl-3H-pyrimidin-4-one* (**3a**). Obtained from **2a** and piperazine (0.86 g, 10 mmol) as a yellow powder, m.p. 215-217 ºC, crystallized from ethanol (74% yield); IR (KBr, cm^−1^): 3,390 (br, NH), 3,035 (CH, aryl), 2,922 (CH, alkyl), 1,690 (CO), 1,631(C=N); ^1^H-NMR: 1.76 (s, 3H, CH_3_), 2.67-2.73(m, 8H, H_piperazine_), 3.74 (s, 3H, OCH_3_), 5.51 (s, 1H, H_pyran_), 6.66 (s, 1H, H_furan_), 6.70 (s, 1H, H_benzene_), 6.90 (s, 1H, H_pyrimidine_), 7.54 (s, 1H, H_furan_), 9.75 (br s, NH, D_2_O exchangeable), 10.25 (br s, NH, D_2_O exchangeable). MS (70 eV, %) *m/z* 407 (M^+^, 88%). Anal. Calc. (Found) for C_21_H_21_N_5_O_4_ (407.42): C, 61.91 (61.88); H, 5.20 (5.10); N, 17.19 (17.22).

*6-(4-Methoxy-7-methylfuro[3,2-g]chromen-5-ylideneamino)-2-morpholin-4-yl-3H-pyrimidin-4-one* (**3b**). Obtained from **2a** and morpholine (0.87 g, 10 mmol) as yellowish crystals, m.p. 203-205 ºC, crystallized from dioxane (76% yield); IR (KBr, cm^−1^): 3,391 (br, NH), 3,034 (CH, aryl), 2,921 (CH, alkyl), 1,688 (CO), 1,629 (C=N); ^1^H-NMR: 1.75 (s, 3H, CH_3_), 3.10 (t, 4H, H_morpholine_) 3.57 (t, 4H, H_morpholine_), 3.72 (s, 3H, OCH_3_), 5.52 (s, 1H, H_pyran_), 6.67 (s, 1H, H_furan_), 6.71 (s, 1H, H_benzene_), 6.92(s, 1H, H_pyrimidine_), 7.53 (s, 1H, H_furan_), 10.20 (br s, NH, D_2_O exchangeable). ^13^C-NMR: 23.3, 48.4, 56.5, 71.8 (6C, CH_3_, 4CH_2_, OCH_3_), 99.9, 101.1, 105.3, 106.9, 108.9, 112.40, 142.3, 145.1, 150.3, 154.2, 155.1, 156.3, 158.5, 160.1 (Ar-C), 169.1 (CO). MS (70 eV, %) *m/z* 408 (M^+^, 89%). Anal. Calc. (Found) for C_21_H_20_N_4_O(408.14): C, 61.76 (61.70); H, 4.94 (4.90); N, 13.72 (13.63).

*6-(4,9-Dimethoxy-7-methylfuro[3,2-g]chromen-5-ylideneamino)-2-piperazin-1-yl-3H-pyrimidin-4-one* (**3c**). Obtained from **2b** and piperazine (0.86 g, 10 mmol) as yellow crystals, m.p. 265-267 ºC, crystallized from benzene (77% yield); IR (KBr, cm^−1^): 3,390 (br, NH), 3,033 (CH, aryl), 2,920 (CH, alkyl), 1,685 (CO), 1,627 (C=N); ^1^H-NMR: 1.73 (s, 3H, CH_3_), 2.65-2.71 (m, 8H, H_piperazine_), 3.76(s, 6H, 2 OCH_3_), 5.54 (s, 1H, H_pyran_), 6.66 (s, 1H, H_furan_), 6.95 (s, 1H, H_pyrimidine_), 7.55 (s, 1H, H_furan_), 9.80 (br s, NH, D_2_O exchangeable), 10.30 (br s, NH, D_2_O exchangeable). MS (70 eV, %) *m/z* 437(M^+^, 86%). Anal. Calc. (Found) for C_22_H_23_N_5_O_5_ (437.45): C, 60.40 (60.35); H, 5.30 (5.39);N, 16.01 (16.21).

*6-(4,9-Dimethoxy-7-methylfuro[3,2-g]chromen-5-ylideneamino)-2-morpholin-4-yl-3H-pyrimidin-4-one* (**3d**). Obtained from **2b** and morpholine (0.87 g, 10 mmol) as a white powder, m.p. 254-256 ºC, crystallized from hexane (75% yield); IR (KBr, cm^−1^): 3,394 (br, NH), 3,031 (CH, aryl), 2,918 (CH, alkyl), 1,680 (CO), 1,625 (C=N); ^1^H-NMR: 1.74 (s, 3H, CH_3_), 3.12 (t, 4H, H_morpholine_), 3.59 (t, 4H, H_morpholine_), 3.75 (s, 6H, 2 OCH_3_), 5.56 (s, 1H, H_pyran_), 6.66 (s, 1H, H_furan_), 6.97 (s, 1H, H_pyrimidine_), 7.52 (s, 1H, H_furan_), 10.26 (br s, NH, D_2_O exchangeable),^ 13^C-NMR: 23.24, 28.2, 56.6, 72.1 (7C, CH_3_, 4 CH_2_, 2 OCH_3_), 99.3, 100.8, 106.9, 108.9, 112.2, 126.5, 140.1, 144.6, 145.4, 148.8, 158.7, 160.2, 162.3, 164.8 (Ar-C), 169.6 (CO). MS (70 eV, %) *m/z* 438 (M^+^, 85%). Anal. Calc. (Found) for C_22_H_22_N_4_O_6_ (438.43): C, 60.27 (60.30); H, 5.06 (5.12); N, 12.78 (12.70).

### 4.5. General Procedure for the Synthesis of (6-Chloro-2-(piperazin/morpholin)-1-yl-pyrimidin-4-yl)-(4-methoxy/4,9-dimethoxy)-7-methylfuro[3,2-g]chromen-5-ylidene)amines **4a-d**

A solution of **3a-d** (10 mmol) in dry dioxane (40 mL) was treated with 10 mL of phosphorus oxychloride, and the mixture was stirred under reflux for 7 h. The reaction mixture was allowed to cool to room temperature, and poured into cold water (100 mL), whereby a solid was separated, filtered off, and crystallized from the appropriate solvent to produce (**4a-d**).

*(6-Chloro-2-piperazin-1-ylpyrimidin-4-yl)-(4-methoxy-7-methylfuro[3,2-g]chromen-5-ylidene)amine* (**4a**). Obtained from **3a** (4.07 g, 10 mmol) as a brown powder, m.p. 281-283 ºC, crystallized from methanol (82% yield); IR (KBr, cm^−1^): 3,380 (br, NH), 3,025 (CH, aryl), 2,915 (CH, alkyl), 1,615 (C=N); ^1^H-NMR: 1.74 (s, 3H, CH_3_), 2.75-2.81 (m, 8H, H_piperazine_), 3.73 (s, 3H, OCH_3_), 5.52 (s, 1H, H_pyran_), 6.65 (s, 1H, H_furan_), 6.71 (s, 1H, H_benzene_), 6.95 (s, 1H, H_pyrimidine_), 7.53 (s, 1H, H_furan_), 9.82 (br s, NH, D_2_O exchangeable). MS (70 eV, %) *m/z* 425 (M^+^, 84%). Anal. Calc. (Found) for C_21_H_20_ClN_5_O_3_ (425.87): C, 59.23 (59.20); H, 4.73 (4.68); N, 16.44 (16.33).

*(6-Chloro-2-morpholin-4-yl-pyrimidin-4-yl)-(4-methoxy-7-methyl-furo[3,2-g]chromen-5-ylidene)-amine* (**4b**). Obtained from **3b** (4.08 g, 10 mmol) as yellow crystals, m.p. 256-258 ºC, crystallized from isopropanol (80% yield); IR (KBr, cm^−1^): 3,020 (CH, aryl), 2,918 (CH, alkyl), 1,617 (C=N); ^1^H-NMR: 1.75 (s, 3H, CH_3_), 3.11 (t, 4H, H_morpholine_), 3.55 (t, 4H, H_morpholine_), 3.72 (s, 3H, OCH_3_), 5.54 (s, 1H, H_pyran_), 6.67 (s, 1H, H_furan_), 6.72 (s, 1H, H_benzene_), 7.1 (s, 1H, H_pyrimidine_), 7.55 (s, 1H, H_furan_). MS (70 eV, %) *m/z* 426 (M^+^, 97%). Anal. Calc. (Found) for C_21_H_19_ClN_4_O_4_ (426.85): C, 59.09 (59.15); H, 4.49 (4.45); N, 13.13 (13.22).

*(6-Chloro-2-piperazin-1-yl-pyrimidin-4-yl)-(4,9-dimethoxy-7-methylfuro[3,2-g]chromen-5-ylidene)-amine* (**4c**). Obtained from **3c** (4.37 g, 10 mmol) as a yellowish powder, m.p. 355-357 ºC, crystallized from dimethylformamide (78% yield); IR (KBr, cm^−1^): 3,391 (br, NH), 3,031 (CH, aryl), 2,922 (CH, alkyl), 1,616 (C=N); ^1^H-NMR: 1.71 (s, 3H, CH_3_), 2.78-2.84 (m, 8H, H_piperazine_), 3.74 (s, 6H, 2 OCH_3_), 5.55 (s, 1H, H_pyran_), 6.67 (s, 1H, H_furan_), 7.12 (s, 1H, H_pyrimidine_), 7.56 (s, 1H, H_furan_), 9.85 (br s, NH, D_2_O exchangeable). ^13^C-NMR: 23.4, 50.3, 56.6, 62.1 (7C, CH_3_, 4 CH_2_, 2 OCH_3_), 99.8, 101.2, 105.2, 106.8, 108.8, 126.4, 140.1, 144.9, 145.2, 148.8, 156.5, 162.4, 164.7, 170.6, 179.8 (Ar-C). MS (70 eV, %) *m/z* 455 (M^+^, 78%). Anal. Calc. (Found) for C_22_H_22_ClN_5_O_4_ (455.89): C, 57.96 (57.90); H, 4.86 (4.81);N, 15.36 (15.32).

*(6-Chloro-2-morpholin-4-yl-pyrimidin-4-yl)-(4,9-dimethoxy-7-methylfuro[3,2-g]chromen-5-ylidene)-amine* (**4d**). Obtained from **3d** (4.38 g, 10 mmol) as a white powder, m.p. 340-342 ºC, crystallized from hexane (76% yield); IR (KBr, cm^−1^): 3,030 (CH, aryl), 2,916 (CH, alkyl), 1,615 (C=N);^1^H-NMR: 1.72 (s, 3H, CH_3_), 3.18 (t, 4H, H_morpholine_), 3.63 (t, 4H, H_morpholine_), 3.74 (s, 6H, 2 OCH_3_), 5.54 (s, 1H, H_pyran_), 6.67 (s, 1H, H_furan_), 7.1 (s, 1H, H_pyrimidine_), 7.53 (s, 1H, H_furan_). MS (70 eV, %) *m/z* 456 (M^+^, 75%). Anal. Calc. (Found) for C_22_H_21_ClN_4_O_5 _(456.88): C, 57.83 (57.79); H, 4.63(4.58);N, 12.26 (12.20).

### 4.6. General Procedure for the Synthesis of ((2,6-di-(Piperazin/morpholin))-1-yl-pyrimidin-4-yl)-((4-methoxy/4,9-dimethoxy)-7-methylfuro[3,2-g]chromen-5-ylidene)amines **5a-d**

To a warm solution of **4a-d** (10 mmol) in methanol (100 mL) was added the freshly distilled piperazine (10 mmol) or morpholine (10 mmol). The reaction mixture was stirred under reflux for 10 h, then allowed to cool to 0 ºC for 12 h. The solid obtained was filtered, washed with water (100 mL), dried, and crystallized from appropriate solvent to produce **5a-d**.

*(2,6-Di-piperazin-1-yl-pyrimidin-4-yl)-(4-methoxy-7-methylfuro[3,2-g]chromen-5-ylidene)amine* (**5a**). Obtained from **4a** (4.25 g, 10 mmol) as yellow crystals, m.p. 261-263 ºC, crystallized from ethanol (85% yield); IR (KBr, cm^−1^): 3,390 (br, NH), 3,030 (CH, aryl), 2,931 (CH, alkyl), 1,620 (C=N); ^1^H-NMR: 1.72 (s, 3H, CH_3_), 2.74-2.80 (m, 8H, H_piperazine_) 3.13-3.19 (m, 8H, H_piperazine_), 3.72 (s, 3H, OCH_3_), 5.54 (s, 1H, H_pyran_), 6.68 (s, 1H, H_furan_), 6.72 (s, 1H, H_benzene_), 6.98 (s, 1H, H_pyrimidine_), 7.55(s, 1H, H_furan_), 9.80-9.95 (br s, 2 NH, D_2_O exchangeable). MS (70 eV, %) 475 (M^+^, 84%). Anal. Calc. (Found) for C_25_H_29_N_7_O_3_ (475.54): C, 63.14 (63.10); H, 6.15 (6.20); N, 20.62 (20.65).

*(2,6-Di-morpholin-4-yl-pyrimidin-4-yl)-(4-methoxy-7-methylfuro[3,2-g]chromen-5-ylidene)amine* (**5b**). Obtained from **4b** (4.26 g, 10 mmol) as brown crystals, m.p. 246-248 ºC, crystallized from ethanol (68% yield); IR (KBr, cm^−1^): 3,031 (CH, aryl), 2,920 (CH, alkyl), 1,622 (C=N); ^1^H-NMR: 1.75 (s, 3H, CH_3_), 2.96-3.02 (m, 8H, H_morpholine_), 3.63-3.69 (m, 8H, H_morpholine_), 3.73 (s, 3H, OCH_3_), 5.56(s, 1H, H_pyran_), 6.66 (s, 1H, H_furan_), 6.70 (s, 1H, H_benzene_), 7.2 (s, 1H, H_pyrimidine_), 7.54 (s, 1H, H_furan_).MS (70 eV, %) *m/z* 477 (M^+^, 86%). Anal. Calc. (Found) for C_25_H_27_N_5_O_5_ (477.51): C, 62.88 (62.80);H, 5.70 (5.68); N, 14.67 (14.55).

*(4,9-Dimethoxy-7-methylfuro[3,2-g]chromen-5-ylidene)-(2,6-di-piperazin-1-yl-pyrimidin-4-yl)-amine* (**5c**). Obtained from **4c** (4.55 g, 10 mmol) as yellow crystals, m.p. 308-310 ºC, crystallized from dioxane (73 % yield); IR (KBr, cm^−1^); 3,395 (br, 2NH), 3,033 (CH, aryl), 2,921 (CH, alkyl), 1,624 (C=N); ^1^H-NMR: 1.72 (s, 3H, CH_3_), 2.73-2.79 (m, 8H, H_piperazine_), 3.13-3.19 (m, 8H, H_piperazine_), 3.75 (s, 6H, 2 OCH_3_), 5.57 (s, 1H, H_pyran_), 6.68 (s, 1H, H_furan_), 7.1 (s, 1H, H_pyrimidine_), 7.53 (s, 1H, H_furan_), 9.83-9.98 (br s, 2NH, D_2_O exchangeable). MS (70 eV, %) *m/z* 505 (M^+^, 93%). Anal. Calc. (Found) for C_26_H_31_N_7_O_4_ (505.57): C, 61.77 (61.70); H, 6.18 (6.13); N, 19.39 (19.32).

*(4,9-Dimethoxy-7-methylfuro[3,2-g]chromen-5-ylidene)-(2,6-di-morpholin-4-yl-pyrimidin-4-yl)-amine* (**5d**). Obtained from **4d** (4.56 g, 10 mmol) as a white powder, m.p. 332-334 ºC, crystallized from hexane (78% yield); IR (KBr, cm^−1^): 3,032 (CH, aryl), 2,920 (CH, alkyl), 1,630 (C=N); ^1^H-NMR (DMSO-d_6_, δ, ppm); 1.73 (s, 3H, CH_3_), 2.97-3.04 (m, 8H, H_morpholine_), 3.69-3.76 (m, 8H, H_morpholine_), 3.73 (s, 6H, 2 OCH_3_), 5.57 (s, 1H, H_pyran_), 6.69 (s, 1H, H_furan_), 7.2 (s, 1H, H_pyrimidine_), 7.52 (s, 1H, H_furan_). ^13^C-NMR: 23.2, 58.8, 56.6, 71.4 (11C, CH_3_, 8CH_2_, 2 OCH_3_), 99.5, 100.2, 103.2, 106.9, 108.9, 126.5, 140.4, 145.1, 146.3, 148.9, 157.5, 161.5, 165.7, 169.7, 177.6 (Ar-C). MS (70 eV, %) *m/z* 507 (M^+^, 82%). Anal. Calc. (Found) for C_26_H_29_N_5_O_6_ (507.54): C, 61.53 (61.57); H, 5.76 (5.70);N, 13.80 (13.85).

### 4.7. General Procedure for the Synthesis of 3-Chloro-1-((4-methoxy/4,9-dimethoxy)-7-methyl-5H-furo[3,2-g]chromen-5-yl)-2,4-dimethyl-6,8-di(piperazin/morpholin)-1-yl-1,3,4,6-tetrahydro-2H-pyrim-ido-[1,6-a]pyrimidines **6a-d**

To a well stirred mixture of **5a-d** (10 mmol) and 3-chloropentane-2,4-dione (1.35 g, 10 mmol) in glacial acetic acid (40 mL), activated zinc dust (10.00 g) was added portionwise at room temperature over a period of 2 h. Stirring was continued for an additional 3 h. Thereafter, the reaction mixture was heated on a water bath (80-90 ºC) for 3 h. The progress of reaction was monitored by TLC. After allowing the reaction mixture to cool to room temperature, it was poured into cold water (100 mL). The insoluble solid which separated was filtered, washed with water, dried and crystallized to produce **6a-d**.

*3-Chloro-1-(4-methoxy-7-methyl-5H-furo[3,2-g]chromen-5-yl)-2,4-dimethyl-6,8-di-piperazin-1-yl-1,3,4,6-tetrahydro-2H-pyrimido[1,6-a]pyrimidine* (**6a**). Obtained from **5a** (4.75 g, 10 mmol) as a yellowish powder, m.p. 350-352 ºC, crystallized from dioxane (82% yield); IR (KBr, cm^−1^): 3,395 (br, 2NH), 3,034 (CH, aryl), 2,932 (CH, alkyl), 1,632 (C=N); ^1^H-NMR: 1.15 (d, 6H, 2CH_3_), 1.73 (s, 3H, CH_3_), 2.63-2.69 (m, 8H, H_piperazine_), 2.83-2.89 (m, 8H, H_piperazine_), 3.14 (m, 2H, H_dihydropyrimidine_), 3.73 (s, 3H, OCH_3_), 3.92 (t, 1H, CH-Cl), 4.60 (s, 1H, H_pyran_), 5.25 (s, 1H, H_pyran_), 6.35 (s, 1H, H_benzene_), 6.67 (s, 1H, H_furan_), 6.83 (s, 1H, H_pyrimidine_), 7.15 (s, 1H, H_pyrimidine_), 7.51 (s, 1H, H_furan_), 9.85, 9.96 (br s, 2NH, D_2_O exchangeable). MS (70 eV, %) *m/z* 582 (M^+^, 80%). Anal. Calc. (Found) for C_30_H_40_ClN_7_O_3_ (582.14): C, 61.90 (61.85); H, 6.93 (6.90); N, 16.84 (16.75).

*3-Chloro-1-(4-methoxy-7-methyl-5H-furo[3,2-g]chromen-5-yl)-2,4-dimethyl-6,8-di-morpholin-4-yl-1,3,4,6-tetrahydro-2H-pyrimido[1,6-a]pyrimidine* (**6b**). Obtained from **5b** (4.77g, 10 mmol) as a yellow powder, m.p. 335-337 ºC, crystallized from methanol (74% yield); IR (KBr, cm^−1^): 3,028 (CH, aryl), 2,918 (CH, alkyl), 1,619 (C=N); ^1^H-NMR: 1.16 (t, 6H, 2CH_3_), 1.72 (s, 3H, CH_3_), 2.99-3.05(m, 8H, H_morpholine_), 3.14 (m, 2H, H_pyrimidine_), 3.66-3.72 (m, 8H, H_morpholine_), 3.75 (s, 3H, OCH_3_), 3.94 (t, 1H, CH-Cl), 4.62 (s, 1H, H_pyran_), 5.24 (s, 1H, H_pyran_), 6.34 (s, 1H, H_benzene_), 6.68 (s, 1H, H_furan_), 6.75 (s, 1H, H_pyrimidine_), 7.30 (s, 1H, H_pyrimidine_), 7.52 (s, 1H, H_furan_). MS (70 eV, %) *m/z* 584 (M^+^, 77%). Anal. Calc. (Found) for C_30_H_38_ClN_5_O_5_ (584.11): C, 61.69 (61.60); H, 6.56 (6.48); N, 11.99 (11.88).

*3-Chloro-1-(4,9-dimethoxy-7-methyl-5H-furo[3,2-g]chromen-5-yl)-2,4-dimethyl-6,8-di-piperazin-1-yl-1,3,4,6-tetrahydro-2H-pyrimido[1,6-a]pyrimidine* (**6c**). Obtained from **5c** (5.05g, 10 mmol) as a brown powder, m.p. 370-372 ºC, crystallized from benzene (72% yield); IR (KBr, cm^−1^): 3,390 (br, 2NH), 3,035 (CH, aryl), 2,924 (CH, alkyl), 1,620 (C=N); ^1^H-NMR: 1.17 (d, 6H, 2CH_3_), 1.76 (s, 3H, CH_3_), 2.70-2.76 (m, 8H, H_piperazine_), 3.15 (m, 2H, H_pyrimidine_), 3.18-3.24 (m, 8H, H_piperazine_), 3.74 (s, 6H, 2 OCH_3_), 3.93 (t, 1H, CH-Cl), 4.62 (s, 1H, H_pyran_), 5.27 (s, 1H, H_pyran_), 6.69 (s, 1H, H_furan_), 6.85 (s, 1H, H_pyrimidine_), 7.20 (s, 1H, H_pyrimidine_), 7.52 (s, 1H, H_furan_), 9.80-9.91 (br s, 2 NH, D_2_O exchangeable). ^13^C-NMR: 22.1, 22.2, 23.1 (3C, 3 CH_3_), 36.5, 45.5, 49.5 (3C, CH), 50.8 (2C, CH_2_), 51.3 (4C,4 CH_2_), 52.3 (2C, 2 CH_2_), 56.7 (2C, 2 OCH_3_), 61.1, 65.2, 84.5 (3C, 3 CH), 101.1, 106.6, 107.1, 108.5, 125.9, 139.5, 142.3, 146.2, 148.7, 150.6, 165.4, 170.8 (Ar-C). MS (70 eV, %) *m/z* 612 (M^+^, 74%). Anal. Calc. for (Found) C_31_H_42_ClN_7_O_4_ (612.16): C, 60.82 (60.89); H, 6.92 (6.97); N, 16.02 (16.10).

*3-Chloro-1-(4,9-dimethoxy-7-methyl-5H-furo[3,2-g]chromen-5-yl)-2,4-dimethyl-6,8-di-morpholin-4-yl-1,3,4,6-tetrahydro-2H-pyrimido[1,6-a]pyrimidine* (**6d**). Obtained from **5d** (5.07 g, 10 mmol) as white crystals, m.p. 385-387 ºC, crystallized from dioxane (69% yield); IR (KBr, cm^−1^): 3,029 (CH, aryl), 2,921 (CH, alkyl), 1,629 (C=N); ^1^H-NMR: 1.16 (d, 6H, 2CH_3_), 1.74 (s, 3H, CH_3_), 2.93-2.99(m, 8H, H_morpholine_), 3.13 (m, 2H, H_pyrimidine_), 3.66-3.72 (m, 8H, H_morpholine_), 3.75 (s, 6H, 2 OCH_3_), 3.95 (t, 1H, CH-Cl), 4.61 (s, 1H, H_pyran_), 5.28 (s, 1H, H_pyran_), 6.67 (s, 1H, H_furan_), 6.88 (s, 1H, H_pyrimidine_), 7.25 (s, 1H, H_pyrimidine_), 7.55 (s, 1H, H_furan_). MS (70 eV, %) *m/z* 614 (M^+^, 78%). Anal. Calc. (Found) for C_31_H_40_ClN_5_O_6_ (614.13): C, 60.63 (60.57); H, 6.56 (6.50); N, 11.40 (11.35). 

### 4.8. General Procedure for the Synthesis of 1-((4-Methoxy/4,9-dimethoxy)-7-methyl-5H-furo[3,2-g]chromen-5-yl)-2,4-dimethyl-3,6,8-tri-(piperazin/morpholin)-1-yl-1,3,4,6-tetrahydro-2H-pyrimido [1,6-a]pyrimidines **7a-d**

To a warm solution of **6a-d** (10 mmol) in methanol (100 mL) was added freshly distilled piperazine (10 mmol) or morpholine (10 mmol). The reaction mixture was stirred under reflux for 12 h, and then allowed to cool to 0 ºC for 12 h. The solid obtained was filtered, washed with water (100 mL), dried, and crystallized from the appropriate solvent to produce **7a-d**.

*1-(4-Methoxy-7-methyl-5H-furo[3,2-g]chromen-5-yl)-2,4-dimethyl-3,6,8-tripiperazin-1-yl-1,3,4,6-tetrahydro-2H-pyrimido[1,6-a]pyrimidine* (**7a**). Obtained from **6a** (5.82 g, 10 mmol) as yellow crystals, m.p. 300-302 ºC, crystallized from ethanol (75% yield); IR (KBr, cm^−1^): 3,398 (br, 3NH), 3,035 (CH, aryl), 2,930 (CH, alkyl), 1,630 (C=N); ^1^H-NMR: 1.12 (s, 6H, 2CH_3_), 1.72 (s, 3H, CH_3_), 2.38-2.44 (m, 8H, H_piperazine_), 2.60-2.66 (m, 8H, H_piperazine_) 2.80-2.86 (m, 8H, H_piperazine_), 3.05 (s, 2H, H_pyrimidine_), 3.1 (s, 1H, H_pyrimidine_), 3.74 (s, 3H, OCH_3_), 4.55 (s,1H, H_pyran_), 5.24 (s, 1H, H_pyran_), 6.33 (s, 1H, H_benzene_), 6.66 (s, 1H, H_furan_), 6.80 (s,1H, H_pyrimidine_), 7.10 (s, 1H, H_pyrimidine_), 7.53 (s, 1H, H_furan_), 9.70, 9.80, 9.90 (3 br s, 3NH, D_2_O exchangeable). MS (70 eV, %) *m/z* 631 (M^+^, 80%). Anal. Calc. (Found) for C_34_H_49_N_9_O_3_ (631.81): C, 64.63 (64.69); H, 7.82 (7.89); N, 19.95 (19.85).

*1-(4-Methoxy-7-methyl-5H-furo[3,2-g]chromen-5-yl)-2,4-dimethyl-3,6,8-tri-morpholin-4-yl-1,3,4,6-tetrahydro-2H-pyrimido[1,6-a]pyrimidine* (**7b**). Obtained from **6b** (5.84 g,10 mmol) as pale yellow crystals, m.p. 296-298 ºC, crystallized from hexane (79% yield); IR (KBr, cm^−1^): 3,029 (CH, aryl), 2,920 (CH, alkyl), 1,623 (C=N) ; ^1^H-NMR: 1.10 (s, 6H, 2CH_3_), 1.73 (s, 3H, CH_3_), 2.36-2.42 (m, 8H, H_morpholine_), 2.93-2.99 (m, 8H, H_morpholine_), 3.04 (s, 2H, H_pyrimidine_), 3.09 (s, 1H, H_pyrimidine_), 3.64-3.70(m, 8H, H_morpholine_), 3.76 (s, 3H, OCH_3_), 4.60 (s, 1H, H_pyran_), 5.25 (s, 1H, H_pyran_), 6.35 (s, 1H, H_benzene_), 6.67 (s, 1H, H_furan_), 6.78 (s, 1H, H_pyrimidine_), 7.20 (s, 1H, H_pyrimidine_), 7.51 (s, 1H, H_furan_). ^13^C-NMR: 21.7, 21.8, 23.2 (3C, 3CH_3_), 36.8, 45.8, 48.5 (3C, CH), 50.4 (2C, 2CH_2_), 51.3 (2C, 2CH_2_), 54.7 (2C, 2CH_2_), 56.7 (1C, OCH_3_), 71.7 (2C, 2 CH_2_), 72.1 (2C, 2 CH_2_), 72.4 (2C, 2 CH_2_), 92.2, 95.8, 99.8, 102.1, 107.2, 107.8, 108.3, 125.5, 139.2, 143.5, 145.2, 148.5, 150.3, 164.4, 169.8 (Ar-C). MS (70 eV, %) *m/z* 634 (M^+^, 82%). Anal. Calc. (Found) for C_34_H_46_N_6_O_6_ (634.77): C, 64.33 (64.28); H, 7.30 (7.35);N, 13.24 (13.30).

*1-(4,9-Dimethoxy-7-methyl-5H-furo[3,2-g]chromen-5-yl)-2,4-dimethyl-3,6,8-tripiperazin-1-yl-1,3,4,6-tetrahydro-2H-pyrimido[1,6-a]pyrimidine* (**7c**). Obtained from **6c** (6.12 g, 10 mmol) as a white powder, m.p. 340-342 ºC, crystallized from dimethylformamide (72% yield); IR (KBr, cm^−1^): 3,394 (br, NH), 3,032 (CH, aryl), 2,925 (CH, alkyl), 1,622 (C=N); ^1^H-NMR: 1.14 (s, 6H, 2CH_3_), 1.74 (s, 3H, CH_3_), 2.37-2.43 (m, 8H, H_piperazine_), 2.62-2.68 (m, 8H, H_piperazine_), 2.82-2.88 (m, 8H, H_piperazine_), 3.08 (s, 2H, H_pyrimidine_), 3.15 (s, 1H, H_pyrimidine_), 3.76 (s, 6H, OCH_3_), 4.58 (s, 1H, H_pyran_), 5.22 (s, 1H, H_pyran_), 6.67 (s, 1H, H_furan_), 6.78 (s, 1H, H_pyrimidine_), 6.92 (s, 1H, H_pyrimidine_), 7.56 (s, 1H, H_furan_), 9.75, 9.85, 9.95 (3 br s, 3 NH, D_2_O exchangeable). MS (70 eV, %) *m/z* 661 (M^+^, 78%). Anal. Calc. (Found) for C_35_H_51_N_9_O_4_ (661.84): C, 63.52 (63.58); H, 7.77 (7.83); N, 19.05 (19.15).

*1-(4,9-Dimethoxy-7-methyl-5H-furo[3,2-g]chromen-5-yl)-2,4-dimethyl-3,6,8-trimorpholin-4-yl-1,3, 4,6-tetrahydro-2H-pyrimido[1,6-a]pyrimidine* (**7d**). Obtained from **6d** (6.14g, 10 mmol) as yellow crystals, m.p. 355-357 ºC, crystallized from dioxane (70% yield); IR (KBr, cm^−1^): 3,030 (CH, aryl), 2,920 (CH, alkyl), 1,627 (C=N); ^1^H-NMR: 1.14 (s, 6H, 2CH_3_), 1.72 (s, 3H, CH_3_), 2.35-2.42 (m, 8H, H_morpholine_), 2.85-2.92 (m, 8H, H_morpholine_), 3.06 (s, 2H, H_pyrimidine _), 3.10 (s, 1H, H_pyrimidine_), 3.64-3.71 (m, 8H, H_morpholine_), 3.74 (s, 6H, 2 OCH_3_), 4.60 (s, 1H, H_pyran_), 5.24 (s, 1H, H_pyran_), 6.68(s, 1H, H_furan_), 6.83 (s, 1H, H_pyrimidine_), 7.10 (s, 1H, H_pyrimidine_), 7.54 (s, 1H, H_furan_). MS (70 eV, %) *m/z* 664 (M^+^, 88%). Anal. Calc. (Found) for C_35_H_48_N_6_O_7 _(664.79): C, 63.23 (63.28); H, 7.28 (7.20); N, 12.64 (12.58).

## 5. Biological Evaluation

### 5.1. Animals

Female Sprague-Dawley rats (150-200 g) were used in the anti-inflammatory activity study. Swiss mice of both sexes weighing 25-30 g were used in analgesic activity tests. International principles and local regulations concerning the care and use of laboratory animals were taken into account. The animals had access to standard commercial diet and water *at libitum* and were kept in rooms maintained at 22 ± 1 °C with a 12 h light-dark cycle.

### 5.2. Anti-Inflammatory Activity (Carrageenan-Induced Rat Hind Paw Edema Model)

The method adopted essentially resembles that described in the literature [17]. Distilled water was selected as vehicle to suspend the standard drugs and the test compounds. Sprague-Dawley rats were starved for 18 h prior to the experiment. The animals were weighed, marked for identification and divided into 28 groups each containing six animals. Edema was induced in the left hind paw of all rats by subcutaneous injection of 0.1 mL of 1% (w/v) carrageenan in distilled water into their footpads. The 1st group was kept as control and was given the respective volume of the solvent (0.5 mL distilled water). The 2nd to 16th groups were orally administered aqueous suspension of the synthesized compounds in dose of 20 mg/kg 1 h before carrageenan injection. The last group (standard) was orally administered diclofenac sodium at a dose of 20 mg/kg as an aqueous suspension [18]. The paw volume of each rat was measured immediately by a mercury plethysmometer, before carrageenan injection and then hourly for 3 h post administration of aqueous suspension of the synthesized compounds. The edema rate and inhibition rate of each group were calculated as follows: Edema rate (E)% = Vt − Vo/Vo, Inhibition rate (I)% =Ec − Et/Ec where Vo is the volume before carrageenan injection (mL), Vt is the volume at t h after carrageenan injection (mL), Ec and Et are the edema rates of the control and treated groups, respectively.

### 5.3. Analgesic Activity Using Hot-Plate Test

The experiment was carried out as described in the literature [19], using a hot-plate apparatus, maintained at 53 ± 0.5 °C. The mice were divided into 28 groups of six animals each. The reaction time of the mice to the thermal stimulus was the time interval between placing the animal in the hot plate and when it licked its hind paw or jumped. The reaction time was measured prior to aqueous suspension of synthesized compounds and drug treatment (0 min). Group 1 was kept as normal control. The aqueous suspension of synthesized compounds was orally administered to mice of groups 2-16 at doses of20 mg/kg. Mice of group 17 (reference) were orally treated with diclofenac sodium at a dose of20 mg/kg body wt. The reaction time was again measured at 15 min and repeated at, 30, 60 and 90 min after treatment. To avoid tissue damage to the mice paws, cut-off time for the response to the thermal stimulus was set at 60 s. The reaction time was calculated for each synthesized compound anddrug-treated group.

### 5.4. Analgesic Activity (Acetic Acid Induced Writhing Response Model)

The compounds were selected for investigating their analgesic activity in acetic acid induced writhing response in Swiss albino mice, following the method described in literature [20]. One hundred and two mice were divided into 28 groups (six in each group) starved for 16 h, pretreated as follows, the 1st group which served as control positive orally received distilled water in appropriate volumes. The 2nd to 16th groups received the aqueous suspension of synthesized compounds orally at a dose of 20 mg/kg. The last group orally received diclofenac sodium at a dose of 20 mg/kg. After 30 min, each mouse was administrated 0.7% of an aqueous solution of acetic acid (10 mL/kg) and the mice were then placed in transparent boxes for observation. The number of writhes was counted for 20 min after acetic acid injection. The number of writhes in each treated group was compared to that of a control group. The number of writhing was recorded and the percentage protection was calculated using the following ratio: % protection = (control mean − treated mean/control mean) × 100.
